# Development and Evaluation of the Personal Patient Profile-Prostate (P3P), a Web-Based Decision Support System for Men Newly Diagnosed With Localized Prostate Cancer

**DOI:** 10.2196/jmir.1576

**Published:** 2010-12-17

**Authors:** Donna L Berry, Barbara Halpenny, Seth Wolpin, B Joyce Davison, William J Ellis, William B Lober, Justin McReynolds, Jennifer Wulff

**Affiliations:** ^6^Swedish Neuroscience InstituteSwedish Medical CenterSeattleUnited States; ^5^University of WashingtonBiobehavioral Nursing & Health SystemsMedical Education & Biomedical InformaticsSeattleUnited States; ^4^University of WashingtonDepartment of UrologySeattleUnited States; ^3^University of SaskatchewanSaskatoonCanada; ^2^University of WashingtonBiobehavioral Nursing & Health SystemsSeattleUnited States; ^1^Dana-Farber Cancer InstituteBostonUnited States

**Keywords:** Prostate cancer, decision making, computer-assisted, pilot study

## Abstract

**Background:**

Given that no other disease with the high incidence of localized prostate cancer (LPC) has so many treatments with so few certainties related to outcomes, many men are faced with assuming some responsibility for the treatment decision along with guidance from clinicians. Men strongly consider their own personal characteristics and other personal factors as important and influential to the decision. Clinical researchers have not developed or comprehensively investigated interventions to facilitate the insight and prioritizing of personal factors along with medical factors that are required of a man in preparation for the treatment decision.

**Objectives:**

The purpose of this pilot study was to develop and evaluate the feasibility and usability of a Web-based decision support technology, the Personal Patient Profile-Prostate (P3P), in men newly diagnosed with LPC.

**Methods:**

Use cases were developed followed by infrastructure and content application. The program was provided on a personal desktop computer with a touch screen monitor. Participant responses to the query component of P3P determined the content of the multimedia educational and coaching intervention. The intervention was tailored to race, age, and personal factors reported as influencing the decision. Prepilot usability testing was conducted using a “think aloud” interview to identify navigation and content challenges. These issues were addressed prior to deployment in the clinic. A clinical pilot was conducted in an academic medical center where men sought consultation and treatment for LPC. Completion time, missing data, and acceptability were measured.

**Results:**

Prepilot testing included 4 men with a past diagnosis of LPC who had completed therapy. Technical navigation issues were documented along with confusing content language. A total of 30 additional men with a recent diagnosis of LPC completed the P3P program in clinic prior to consulting with a urologist regarding treatment options. In a mean time of 46 minutes (SD 13 minutes), participants completed the P3P query and intervention components. Of a possible 4560 items for 30 participants, 22 (0.5%) were missing. Acceptability was reported as high overall. The sections of the intervention reported as most useful were the statistics graphs, priority information topics, and annotated external website links.

**Conclusions:**

The P3P intervention is a feasible and usable program to facilitate treatment decision making by men with newly diagnosed LPC. Testing in a multisite randomized trial with a diverse sample is warranted.

## Introduction

There is a growing body of evidence that men with a recent diagnosis of localized prostate cancer (LPC) conduct the treatment decision-making process by strongly considering their own personal characteristics and other personal factors [1-7]. Recently diagnosed men must often assume some responsibility for the treatment decision together with guidance from clinicians. The participation of patients with cancer in making decisions about treatment is promoted by virtually all interested parties including professional societies, researchers, and clinicians. Information widely available via the Internet, though of variable accuracy, has helped to hasten a new dynamic between patient and clinician. Because no other disease with the high incidence of prostate cancer has so many alternative treatments with so few certainties related to outcome, many men are faced with assuming some responsibility for the treatment decision along with guidance from clinicians.

Yet clinical researchers have not comprehensively investigated interventions to facilitate the gaining of insight and the prioritizing personal factors as well as the decision making that are required of a man with a diagnosis of LPC. Decision support technologies provide much needed information to patients, but (1) focus solely on medical factors considered relevant by physicians (eg, histology, comorbidity, and age), (2) fail to customize the information to the personal characteristics of the patient [8-10], or (3) depend on interventions that have never been rigorously tested in randomized trials with diverse samples [2,11,12]. The goal of this ongoing program of research is to improve the decision-making experience for men with LPC by highlighting personal characteristics and factors that men bring to the treatment decision: their desired level of participation in decision making, the importance of potential outcomes and complications, current symptoms, priority information topics, the influence of others, race/ethnicity, and self-perception of age.

This research was informed by O’Connor’s Decision Support Framework (DSF) [13]. The framework is most appropriate for health care situations in which careful deliberation is required because of many uncertainties and value-sensitive risk/benefits and for which the deliberation phase (deciding) requires substantially more effort than the implementation phase (undergoing a particular management strategy or therapy). The DSF is organized by (1) determinants of decisions, (2) decision support interventions, and (3) evaluation of both the process and outcomes of the decision support. Since our research team had documented the determinants, that is, the personal factors brought to the decision by men with LPC [1,7], we were poised to engage in the second step, designing the support intervention and evaluating the process.

In this paper, we report the iterative development and initial evaluation of a tailored Internet patient decision support system, the Personal Patient Profile-Prostate (P3P), in which all of these factors are assessed and addressed. The research aims included the following system requirements: (1) specifications of use cases, (2) application architecture and content, (3) usability, and (4) feasibility.

## Methods

### Preclinical Design Overview

An iterative development approach was employed, beginning with the development of use cases (see Multimedia Appendix 1) by members of the research team. Within these cases and through contextual inquiry with potential users and investigators (including content and informatics experts), we used a structured process for identifying end users, requirements, and application content. In addition to gathering end user requirements, we closely adhered to National Cancer Institute’s recommendations about appropriate user interface design [14]. The application was initially implemented in 2004. Detailed methods are presented below with respect to the preclinical design phase, with methods grouped by architecture, query, and intervention content.

### Application Architecture

The application architecture for P3P utilizes an open source Web software platform and provides for a flexible survey environment [15]. The survey environment also enables the overlay of interventional content The design employs a modular, extensible approach built on the generalized storage and display of survey instruments. All survey content associated with a specific instrument is stored in a database, and “assessments” can be compiled from multiple survey instruments. Each survey instrument is represented as a reusable object containing questions, possible answers, and control logic. The software retrieves these objects and displays them to the user, recording answers as well as metadata such as time stamps and navigation information. A survey editor allows researchers to make changes to the content and the sequence of questions without software modifications. The survey framework also includes a patient manager to enter patient demographics and manage the patient data associated with a specific administration of an assessment in a clinical setting.

The Web application was implemented using the PHP language and MySQL database on a Linux/Apache server, a development platform commonly known as "LAMP” [16]. All user interface components were implemented as dynamic server-side pages. Surveys were presented using templates, making it simple to adapt the system to conform to user interface guidelines and to a variety of device characteristics. Touch screen monitors were utilized to display and access the P3P program. This hardware, along with the navigational design of the program, eliminates the use of a mouse and scrolling. Keyboarding is not required but is available for optional open-ended items. User interface widgets (radio buttons, checkboxes, and navigation buttons) can be resized to work well with touch screens via a simple configuration option.

### Application Content

#### Profile Query Component

The purpose of the profile query was to gather the input necessary for the tailored intervention component. The P3P opening screen introduced the participant to the nature and purpose of the intervention, that is, to help make “the best choice for you,” providing information and guidance to understand the participant’s personal concerns. In addition to demographic characteristics, the following valid and reliable instruments were presented in the query component of P3P in order to generate the intervention (Table 1). All instruments were adapted for the touch screen by presenting 1 item per screen, with the exception of some items from the Expanded Prostate Cancer Index Composite Short Form 6.2002 (EPIC-SF) that were logically presented next to each other (see below). Additional questionnaires were presented to the patient during the query component which were outcomes of using the P3P tailored intervention in a future randomized trial, and we thought best to pilot test the inclusion of instruments to measure state anxiety [17] and decisional conflict [18]. Finally, we included an acceptability assessment [19]. Reading grade level of the application’s internal content was calculated using the Flesch-Kincaid measure for an average of 7.6 (SD 1.6) and ranging from 5.4 to 10.0. Skipping questions without answering was allowed.

**Table 1:** Variables and questionnaire results used to generate the P3P tailored intervention

Variable	Questionnaire
Sociodemographic characteristics	Demographic data form
Influential personal factors	Personal Profile [7]
Information preferences	Patient Information Program [20]
Decisional control	Control Preferences Scale [21]
Symptoms	EPIC-SF [22]

##### Personal Profile

The Personal Profile was developed by the investigators based on the earlier qualitative work [1] and was designed for and used with 260 men during a descriptive quantitative study of the personal factors that influence men’s treatment decisions [7]. Face validity and test-retest reliability of the Personal Profile have been established [7]. The profile contains ranking of the following personal factors with regard to influence on the decision or importance to the decision: influential people (spouse/partner, family member, coworker, friend, and celebrity), influential outcomes (bladder, bowel and sexual function, and expected survival) and personal characteristics (confidence in the doctor, age, work, and recreational activities). Each of these item responses was listed as “no influence,” “a little influence,” “some influence,” or “a lot of influence.”

##### Control Preferences Scale and Information Priorities

The Control Preferences and Informational Priorities were the 2 components of the Patient Information Program (PIP) developed by Davison and colleagues [20]. The first component of the PIP uses the Control Preferences Scale modified by Davison [23] to elicit patients’ preferences for control over treatment decision making. The second component of the PIP focuses on identifying priority information topics and is based on a paper and pencil survey previously developed and validated by Davison in samples of men newly diagnosed with prostate cancer.

##### Expanded Prostate Cancer Index Composite Short Form 6.2002

Prostate cancer-targeted symptoms were assessed using the 4 prostate-targeted symptom domains developed by Wei and his colleagues [22]: Sexual, Hormonal, Urinary, and Bowel. Each scale of the EPIC-SF includes a function subscale and a “bother” item. In P3P, the final item of the EPIC-SF, assessing the patient’s perception of “how big a problem” for 5 hormonal symptoms, was displayed in a matrix on 1 screen.

#### Outcome Measures (Did Not Create an Intervention Component)

##### Anxiety

Anxiety was measured with the 20-item State component of State-Trait Anxiety Inventory (STAI) [17], which is also known as the Self-Evaluation Questionnaire.

##### Decisional Conflict

The Decisional Conflict Scale (DCS) [18] measured the conflict inherent in the treatment decision encountered by the men in the sample. Of the subscales of the DCS, 2 (Uncertainty and Factors-Contributing-to-Uncertainty) are appropriate for use before or during decision-making and the third (Effectiveness of Decision Making) for use after the decision has been made.

#### Intervention Component

Immediately after a participant had completed the query component, the P3P intervention was delivered to him in 5 distinct sections. First, the participant was shown a screen that listed the levels of decisional control preference with the earlier selected level highlighted. The participant was then instructed to play the video clip matching the selected control level. Next, the 4 priority categories of “information needed today” that were ranked highest in the Patient Information Program component were displayed as brief narrative text on-screen summarizing the priority topic, and a full page teaching sheet was printed for each topic. Third, a statistics tutorial was displayed utilizing the highest ranked influential outcome: survival, bladder, bowel, or sexual function. The screen included explanatory text and an exemplar percentage chart such as 17 frowning faces and 83 smiling faces in a rectangular matrix of 100 faces.

The fourth section began with a screen containing a menu of topics covering the influential factors that had been ranked as having some or a lot of influence on treatment decision making. When viewed, each topic included a brief narrative description of the issue and a corresponding video clip depicting a patient discussing the topic with a physician. Finally, an option to explore 4 reputable, informational prostate cancer websites [24] to which an annotated guide and links were provided was presented as the final intervention component. The clinicians on this investigative team reviewed the websites for current information.

Table 2 summarizes how the intervention was tailored to the patient’s personal profile and provides links to screenshots in the Multimedia Appendices.

**Table 2:** P3P intervention customization by the patient’s personal profile

Patient Query Component	Internal Algorithm	Intervention Delivered to Patient
Prostate cancer information priorities:	Patient was presented 36 paired comparisons of 9 information topics and selected from each pair the topic of greater priority to him to receive information The top 4 most highly prioritized were calculated.	Information relevant to the top 4 priorities was briefly summarized on-screen. At the end of the intervention, the patient received printed teaching sheets on each topic.
Stage of disease Prognosis Treatment options Side effects Home self-care Impact on family Sexuality Social activities Family risk		
Demographics:	Patients’ ages were categorized as under 60 or 60+ years of age. Patients self-identified as white, black, or other (Asian, Native American).	Videos featured a patient actor close to the patient in age and matched for race as below; those reporting “other” or skipping the race item were offered intervention content tailored to white patients.
Date of birth Self-reported race		
Preferred role in the Treatment decision (Control Preferences Scale)	Patient selected response option: 1 or 2 (active role) 3 (shared role). 4 or 5 (passive role) The preferred role was highlighted in the intervention text and video, (Multimedia Appendix 2).	Text and video coaching customized to patient’s race was offered for a patient to express his preferred role. In the video, the doctor acknowledged the patient’s preference (Multimedia Appendix 3). The patient was offered the opportunity to view the text and video for other control preferences.
Influential People:	Patient selected option for how much influence these people had as he considered his treatment choices: (1) no influence (2) a little influence (3) some influence (4) a lot of influence. For each reported to have “some influence” or “a lot of influence,” the intervention offered text and a video coaching the patient to tell his doctor.	Text and video coaching were offered for the patient to express who were the influential people in his decision process. The doctor in the video acknowledged the importance of these influential people and helped the patient compare his own views and situation to those of influential people (Multimedia Appendix 4). At the end of the intervention, the patient printed the teaching information with “fill in the blank” text he could use to prepare for the exam visit.
coworkers friends outside work spouse/partner other family members		
Influential outcomes:	For each of these treatment outcomes, the patient selected how much importance or influence it had on his decision: (1) no influence (2) a little influence (3) some influence (4) a lot of influence. The outcome rated most influential was used as the example for teaching about statistics. In the case of a tie between outcomes, the example was selected randomly from those rated most highly influential. For the outcomes rated “some influence” or “a lot of influence,” the patient was offered text and video coaching.	Text and a graphic illustration taught numeracy skills useful to understanding statistics about possible outcomes. The example provided was highly salient to the patient (Multimedia Appendix 5). Text and video coaching customized to age was offered for the patient to express the influential factors in his decision process. The doctor in the video acknowledged the importance and helped the patient understand the relative likelihood of each treatment option’s impact on these factors (Multimedia Appendix 6). At the end of the intervention, the patient printed the teaching information with “fill in the blank” text he could use to prepare for the exam visit.
survival bladder function bowel function sexual function		

Current symptoms: (EPIC questionnaire)	Each symptom domain included an overall impact item. For each item where the patient responded that the symptom is a “moderate problem” or a “big problem,” the symptom was listed on the intervention menu page to learn more about (Multimedia Appendix 7).	Text and video coaching customized to race was offered on each symptom the patient experiences as a problem. In the video, the patient reported his symptom and the doctor offered to help him understand how different treatments might impact his symptoms differently (Multimedia Appendix 8). At end of the intervention, the patient printed the teaching information with “fill in the blank” text he could use to prepare for the exam visit.
urinary bowel sexual		
Useful links	Not customized–the same content was offered to all patients.	Links to 4 highly rated professional websites offering general information about prostate cancer.

#### Acceptability

At the completion of the program, aspects of patient acceptability (easy, understandable, enjoy, helpful, time, value of information, and overall satisfaction) were measured for the entire P3P with the Acceptability E-scale, a 1 to 5 scale anchored by 1 = not easy at all (for example) to 5 = very easy (for example) [19]. We added 1 item, “value of information,” to our previous scale. An additional set of investigator-developed usefulness queries was presented using a similar 1 to 5 scale and focused on the unique sections of the intervention.

### Prepilot Usability Testing in Proxy Patients

The University of Washington Human Subjects Division approved all study procedures and materials. In all, 4 English-speaking men who were at least 6 months post-prostate cancer treatment were recruited from a university-based prostate cancer clinic practice to test the usability of the P3P program. Each consenting participant was asked to complete the P3P prototype using a touch screen monitor and desktop computer in an informatics laboratory at the University of Washington, School of Nursing. Simultaneously, an audio-recorded cognitive clinical interview [25], also known as the “think-aloud” method [26], was conducted by a graduate nursing student (author JW). The purpose of this usability testing was to asses how men interacted with the technology and to identify problems with the interface and/or content. The subjects were asked to “think-aloud” as they went through the questions and the intervention on-screen in order to understand their experience of the system.

### Clinical Pilot Testing

**Figure 1:**
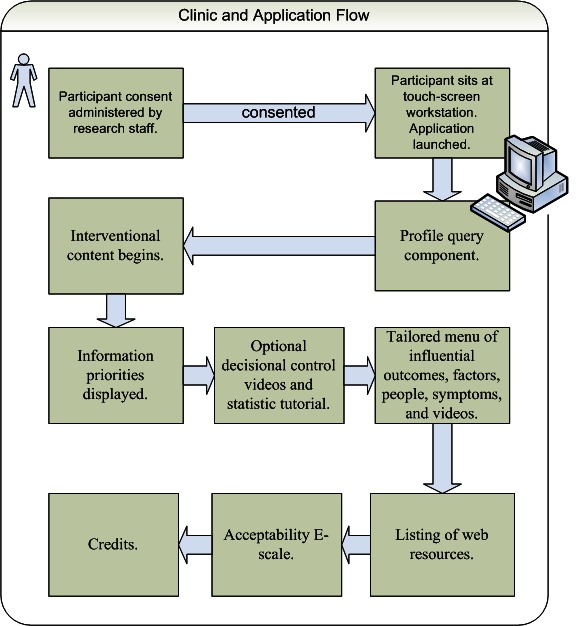
Clinical and application flow

A total of 32 English-speaking men with newly diagnosed LPC who sought consultation at the University of Washington Medical Center’s Prostate Oncology Center were invited to participate by clinic nursing staff. Of these, 30 men provided informed consent and were enrolled by a research team member. Participants used the program on a touch screen monitor with a full-size keyboard connected to a desktop computer in the center’s patient education room. The team member was waiting in an adjacent room and available for assistance if needed and made summary notes of each participant’s session including any usability issues reported, feedback offered, and whether a spouse or partner viewed the program with the participant. The participant then proceeded to the consult visit with 1 or more prostate cancer specialty physicians within an hour of using the intervention. Figure 1 displays the clinical and application flow.

### Analysis

Usability testing data from the prepilot were analyzed descriptively with quantitative content analysis [27]. In addition, the following quantitative measures were used for the clinical pilot results: completion time, data completeness, and acceptability scores. The questionnaire results will be reported elsewhere.

## Results

### Prepilot

The program and cognitive interview took 1 to 1½ hours to complete. The interviews were audio-recorded and transcribed verbatim. Table 3 lists the tasks and observations and responses from the testing. Minor edits were made to the P3P program based on these formative findings.

**Table 3:** Summary of participants’ responses and observations during prepilot usability testing (N=4)

Goals	Task	Observations and Responses Regarding Content and Technical Aspects of the Program (n for Each Observation)
Overall ease of use	Page navigation: Understand and follow navigation instructions	Technical: Expectation of auto advance vs use of “next” button (3) Progress bar meaning unclear (3) Dislike of required scrolling on certain screens (due to resolution of personal computer monitor used in testing) (2)
Complete query component	Understand and answer each item of each scale	Content: Confusion between “ethnicity” and “race” (2) Doubt was expressed regarding the ability of most patients to identify a treatment preference after the biopsy and before the appointment to review options (3) Request for brief explanation of treatment options before query component (1) The phrase “home self-care” confusing (3) Wording of information priority pairings led participants to believe the same item was repeated (4) Difficulty recalling which section was which when asked to evaluate the various sections (4)
Receive customized education/coaching component	Open, understand, and review sections	Technical: Unclear how to proceed after video clips (4) Double clicks used to open menu items when single clicks were adequate (1) Content: Information and content valuable (4)

### Clinical Pilot

Of the 30 participants, 26 (87%) completed query and intervention components of the P3P program in less than 1 hour (mean 46 minutes, SD 13 minutes, range 16 to 69 minutes). The sample had a mean age of 61, ranging from 45-74. Missing data were minimal: 15 participants answered every one of the 152 items, 8 men skipped 1 item (0.6%), and 7 men skipped 2 items (1.3%). Of a possible 4560 items for 30 participants, 22 (0.5%) were missing. All participants watched the tailored video clip describing the identified decision control preference, and 7 men watched additional clips representing other control preferences. In all, 10 men viewed a video clip from the menu of personal factor results. The majority, 28 of the 30 men, viewed at least 1 set of text and graphic statistics tutorials about survival, incontinence, impotence, or bowel disturbance.

Overall acceptability of P3P was calculated as high (Table 5). At least 67% of the sample chose a 4 or 5 on each of the acceptability items

Participants reported on the usefulness of the specific intervention components viewed (Table 6). Over half of all participants who viewed each component reported usefulness at 4 or 5 on the 5-point scale. In all, 5 participants did not have time to access or chose not to view the additional websites.

There were no significant relationships between age, education, or work status and any usefulness outcome (data not shown). Many verbal questions/comments were made by participants regarding future home access to the program. Many participants also said that the information would have been more useful a few days prior to the consult with the urologist. Participants reported perceived time pressure in the clinic to finish viewing all components before being called in to see the physician. Additional comments were written by 6 men; of these, 5 offered critiques of wording or display in the program, and 1 man endorsed the content on family impact.

Research team member notes were available for 23 of 30 sessions and indicated 2 usability problems: 1 participant was unsure how to navigate away from an informational prostate cancer website pop-up linked from the intervention, and another did not know how to print teaching sheets at the end of the program. An additional 3 men were noted to have trouble hearing the videos. Also, 13 men were reported to have viewed the intervention together with a spouse or partner, while 10 did not.

**Table 4:** Demographic characteristics of participants (N = 30)

		n	%
**Ethnicity**			
	Hispanic/Latino	0	0
	Missing	2	6.7
**Race**			
	White/Caucasian	29	96.7
	American Indian/Native Alaskan	1	3.3
Married/partnered		24	80
**Work status**			
	Working (full-time or part-time)	17	56.6
	Not working (retired or unemployed)	13	43.3
**Annual household income**			
	≤ US $35,000	4	13.3
	US $35,001-55,000	4	13.3
	US $55,001-85,000	7	23.3
	≥ US $85,001	15	50.0
College graduate		23	76.7
Home Internet access		29	96.7
Frequent computer user		25	83.3
**Primary health insurance**			
	Private	22	73.3
	Medicare	7	23.3
	Missing	1	3.3
**Number of weeks since biopsy**			
	< 4	9	30.0
	4 and over	21	70.0

**Table 5:** Overall P3P Acceptability (N=30)

Item	Mode	Mean (SD)
Easy to Use	5	4.8 (.41)
Understand questions	5	4.7 (.52)
Time to complete	5	4.5 (.78)
Enjoy program	5	4.0 (.98)
Helpfulness of program	5	4.0 (1.0)
Value of Information	4	3.7 (1.0)
Overall satisfaction	5	4.1 (.92)

**Table 6:** P3P intervention component usefulness

Component	n	Mode	Mean (SD)
Statistics graphs	30	5	3.8 (1.6)
Control preference graph	30	3	3.8 (1.1)
Priority information topics	29	5	3.7 (1.3)
Video clips	30	3	2.8 (1.2)
Websites	25	5	4.0 (1.2)

## Discussion

The P3P was successfully developed, tested, and deployed in an academic medical center urology clinic by an interdisciplinary research team. The acceptability and usefulness scores plus verbal and written comments have given us areas with which to further revise and develop the program.

There was a high level of interest in participating. Travel logistics were cited by the 2 men who did not participate as the primary reason for not enrolling. This problem together with comments requesting access to the program a few days before the options review visit indicated a strong need for home access to the intervention.

Men may also have been more likely to view the menu items with which they were the least familiar, notably statistics. Only 10 men were able to, or chose to, view the video clips providing coaching on how to speak with one’s doctor regarding influential personal factors. We are uncertain as to why the other 20 men did not view the video clips although this could be explained by lack of time or misunderstanding of the screen instructions or menu display. The men may have been constrained by time at the completion of the program since they immediately went into their clinic visit with the urologist. There is some evidence from a subsequent cognitive interview study of African American men using P3P [28] that the navigation of the menu display for the video clips was not readily apparent.

Methods of evaluation applied in this study have been used by other health technology evaluations of tailored intervention in 15 healthy women relevant to preventing osteoporosis [29] and 13 Hispanic family caregivers relevant to health promotion [30]. Findings of all these trials indicate that performance usability testing in settings that mirror the intended use setting can successfully identify areas and functions of the applications that require modification. Patient-centered decision support technologies posted on the Internet or provided in larger electronic health systems that have not been exposed to rigorous usability testing are inherently suspect for poor generalizability and potential end user dissatisfaction at best, or poor uptake and disuse, at worst.

The reported acceptability of this tailored decision aid is comparable to that of other electronic, Web-based applications developed by this research team and colleagues. The electronic self-report assessment for cancer (ESRA-C) is a screening assessment for patients with all cancer diagnoses shown to be efficacious for improving clinician patient communication [31]. Wolpin et al [32] reported similarly high levels of acceptability for the ESRA-C application including 342 ambulatory patients with cancer who had completed the program at 2 time points. Intervention websites in health care have included quantitative acceptability measures typically developed by the researcher as study-specific scales or item sets and which have not been validated. For example, DiLorio and colleagues [33] reported the success of a Web-based self-management program for epilepsy from participants’ perspectives with regard to overall satisfaction and component-specific satisfaction. While we were unable to compare the scores because the instruments varied somewhat, the approach was feasible in both studies and both differentiated satisfaction with various program components. Furthermore, our scale for user self-report of satisfaction and acceptability has been tested for reliability and dimensionality. Tariman et al [19] analyzed a sample of 627 respondents and reported that the Acceptability E-scale was found to have a consistency coefficient of .76, good item-to-item and item-to-scale correlations, and was unidimensional.

A large team of researchers recently developed the International Patient Decision Aid Standards Collaboration instrument (IPDASi) [34] as a means to evaluate decision support technologies. While the P3P contains each of the 9 applicable dimensions, a quantitative scoring of P3P has not been conducted using the IPDASi.

Our pilot findings are limited by the evaluative scope of the study, namely feasibility, usability, and satisfaction with a new decision support system. Our participants were fairly well educated with most household incomes in the 4^th^ or 5^th^ quartile for the region and predominately white, precluding generalization beyond this group of men. However, the results have guided the redesign and deployment of P3P for testing in a multisite, randomized trial with a diverse sample [35]. The new application is accessible from remote (home) locations on varied hardware and software.

In conclusion, our preliminary evidence suggests that the P3P is a useful and acceptable decision support system that feasibly can be deployed in a clinical practice setting. The program enables men with early stage prostate cancer to identify and understand the personal issues and factors that influence a treatment decision and coaches men to articulate those issues and factors to the consulting physician.

## Multimedia Appendix 1

Personal Patient Profile-Prostate (P3P): Use case in urology

## Multimedia Appendix 2

Screenshot of CPS video highlighted for patient’s selection

## Multimedia Appendix 3

Decisional control video: Active participation

## Multimedia Appendix 4

Influential people video: Coworker

## Multimedia Appendix 5

Screenshot of a statistics teaching text/graphic page

## Multimedia Appendix 6

Influential outcome video: Bladder

## Multimedia Appendix 7

Screenshot of an EPIC urinary domain item

## Multimedia Appendix 8

Screenshot of a current symptom coaching page

## Abbreviations

DSF: Decision Support Framework
ESRA-C: electronic self-report assessment for cancer
EPIC-SF: Expanded Prostate Cancer Index Composite Short Form 6.2002
IPDASi: International Patient Decision Aid Standards Collaboration instrument Personal
PIP: Patient Information Program
P3P: Personal Patient Profile-Prostate
