# Supplementary material for: Development and Evaluation of the Personal Patient Profile-Prostate (P3P), a Web-Based Decision Support System for Men Newly Diagnosed With Localized Prostate Cancer
Source: J Med Internet Res. 2010 Dec 17;12(4):e67. doi: 10.2196/jmir.1576 (PMC3056527; doi:10.2196/jmir.1576)
Supplement: Supplementary file 1 [file jmir_v12i4e67_app1.pdf]

Multimedia Appendix 1. Personal Patient Profile-Prostate (P3P): Use Case in Urology

- Urologist calls patient with localized prostate cancer to tell him results of biopsy and initiate the next step (typically an appointment for the options talk. During the call, the urologist will say, “We are developing a new way to help prepare you for the treatment option discussion using a computer program that is designed to be user-friendly. It may help you identify your particular concerns and help you understand more about the issues involved in the decision. If you’ll take a look at it, I’ll have one of the research team members set this up for you in the clinic.” If he is interested in hearing more, the urologist tells him that the study coordinator will call. The urology Patient Care Coordinator (PCC) will have scheduled an appointment for the options talk. The study coordinator (SC) calls within 48 hours and explains more about the study. If the patient is interested, he is asked to come to clinic an hour before his next scheduled appointment.
- The patient arrives an hour before the appointment. The SC greets the patient and takes him to the education room. The study is fully explained and written consent is obtained. The SC logs in the PC and identifies the patient with the medical record number. The opening screen is visible when the patient is seated at the PC. The SC explains that the monitor is a touch screen and that to us it, he only has to touch the appropriate place. The patient is provided with a cotton swab to use as a stylus and he is told he also can use his fingertip or the mouse. The SC gives him the earphone set that is already plugged into the PC and assists him to put it on. If the patient refuses the earphone set, the PSR

must unplug the set from the PC. The SC withdraws from the room, offering to be nearby for help if needed.

- Continuing past the opening screen, an orientation screen appears, telling the patient how to navigate in the program. He then is presented with a series of questionnaires. After completing the “query” component, the intervention component begins without a prompt.   The participant has the opportunity to view specific video clips modeling interactions between patients and physicians around the issues he identified, read about statistics, read the results of his information priorities ranking, read about level of participation in the decision, read about potential complications and finally, link to reliable, outside Internet resources. If he chooses none of the options he is automatically shown the results of the information priorities, the decision control preference; a summary of influential personal factors plus suggested discussion topics to address with the clinician. He has the opportunity at this point to correct any ranking. At the conclusion of the session, he is asked to answer a set of acceptability questions. The final screen instructs him to return to the SC.
- The patient goes in to see the urologist
