# Supplementary figures and images for: Development and Evaluation of the Personal Patient Profile-Prostate (P3P), a Web-Based Decision Support System for Men Newly Diagnosed With Localized Prostate Cancer
Source: J Med Internet Res. 2010 Dec 17;12(4):e67. doi: 10.2196/jmir.1576 (PMC3056527; doi:10.2196/jmir.1576)

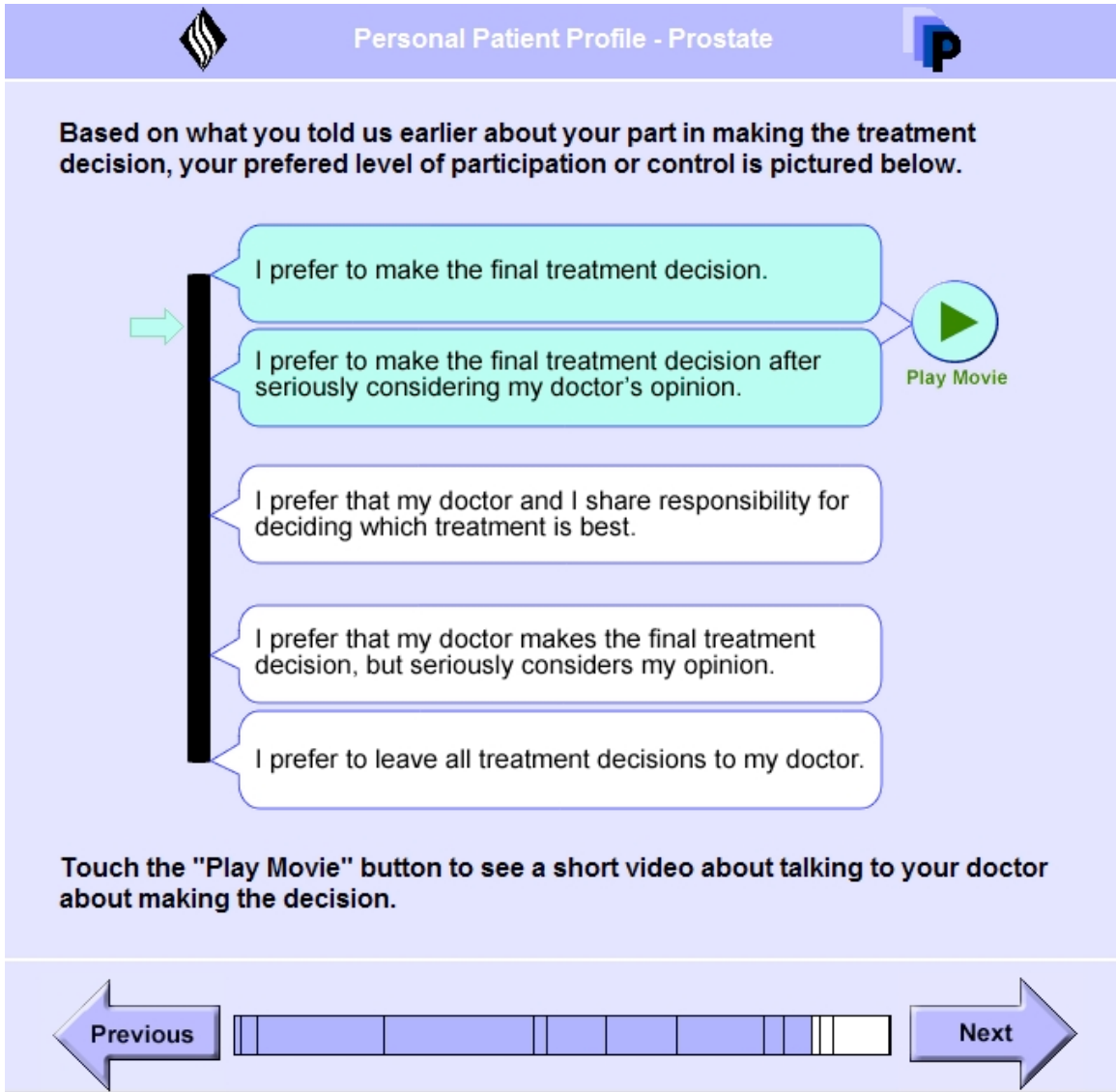

Supplement: Supplementary file 2 [file jmir_v12i4e67_app2.pdf]

**Multimedia Appendix 4.** Influential people video: Co-worker

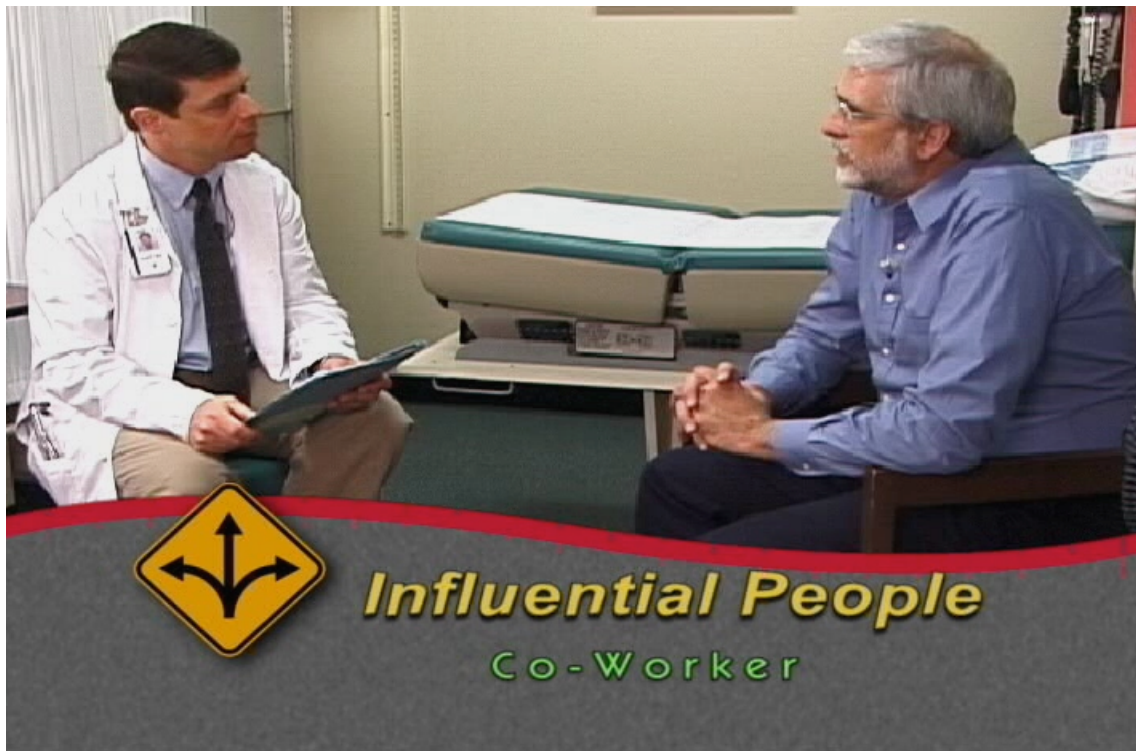

Supplement: Supplementary file 5 [file jmir_v12i4e67_app4.pdf]

**Multimedia Appendix 6. Influential outcome video: Bladder**

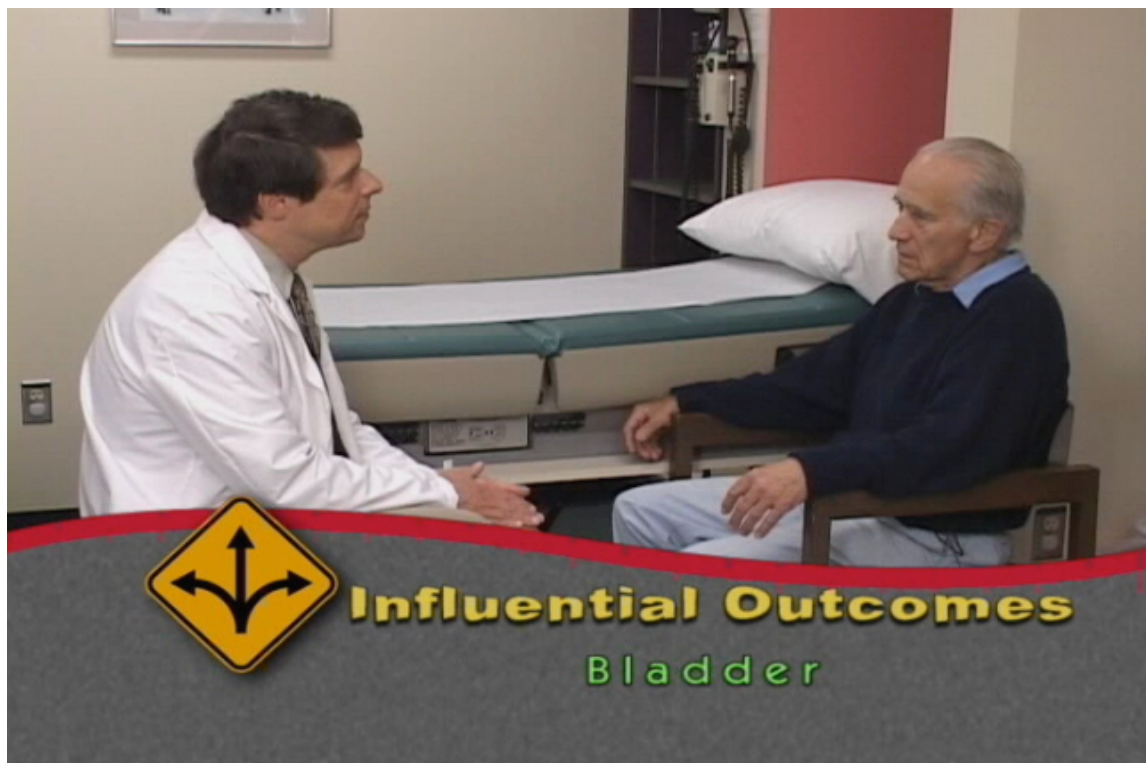

Supplement: Supplementary file 8 [file jmir_v12i4e67_app6.pdf]
