# Supplementary material for: Development and Evaluation of the Personal Patient Profile-Prostate (P3P), a Web-Based Decision Support System for Men Newly Diagnosed With Localized Prostate Cancer
Source: J Med Internet Res. 2010 Dec 17;12(4):e67. doi: 10.2196/jmir.1576 (PMC3056527; doi:10.2196/jmir.1576)

Multimedia Appendix 5. Screenshot of a statistics teaching text/graphic page

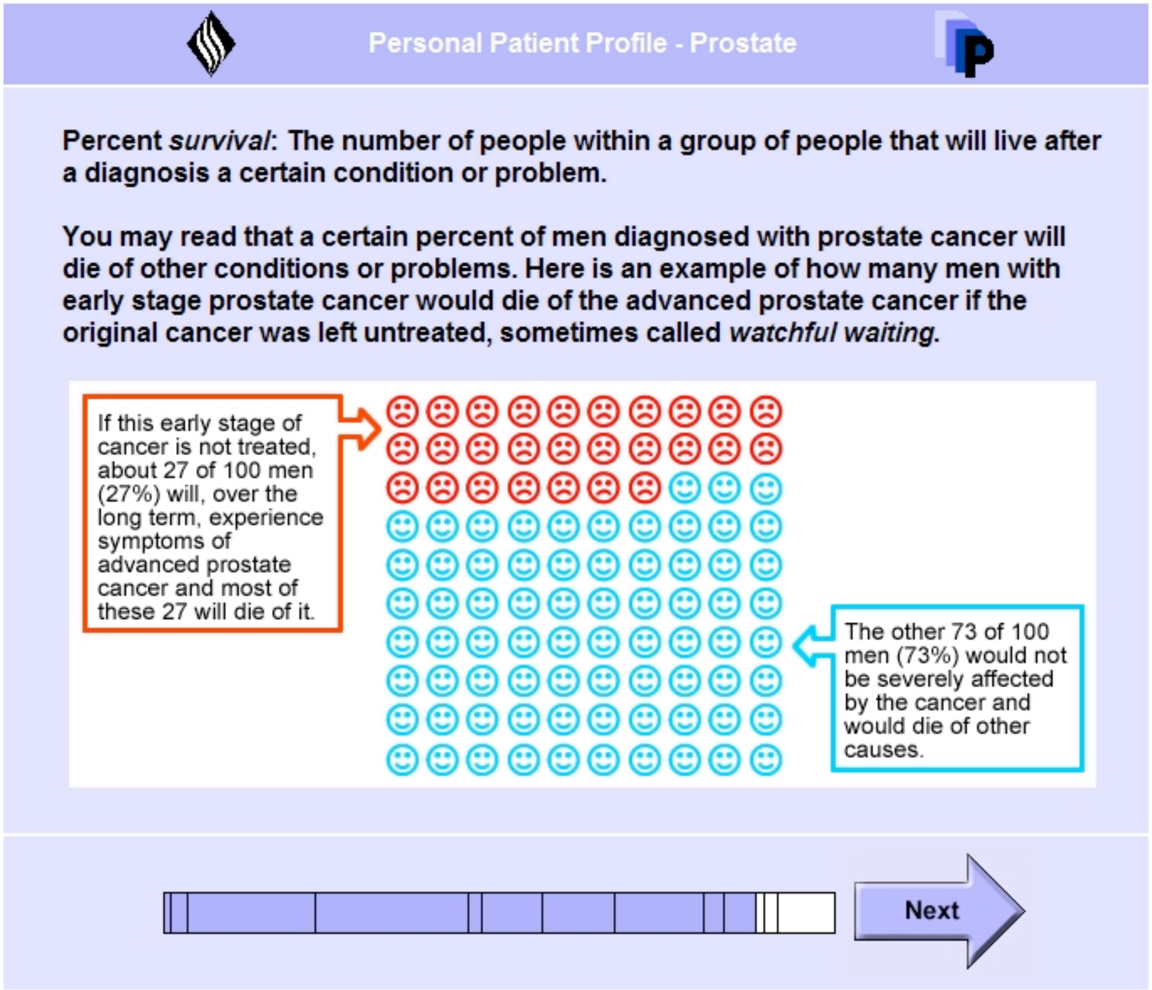

Supplement: Supplementary file 7 [file jmir_v12i4e67_app5.pdf]
